# Supplementary material for: Robotic scrub nurse to anticipate surgical instruments based on real-time laparoscopic video analysis
Source: Commun Med (Lond). 2024 Aug 2;4:156. doi: 10.1038/s43856-024-00581-0 (PMC11297199; doi:10.1038/s43856-024-00581-0)
Supplement: Supplementary file 2 — Supplementary Information [file 43856_2024_581_MOESM2_ESM.pdf]

Supplementary information

		N
**Gender**	Female	17 (34%)
	Male	33 (66%)
	Diverse	0 (0%)
**Age**	18 - 33 years	20 (40%)
	34 - 44 years	17 (34%)
	45 - 55 years	9 (18%)
	56 - 65 years	3 (6%)
	> 65 years	17 (34%)
**Position**	Resident	21 (42%)
	Attending	14 (28%)
	Senior physician	12 (24%)
	Department head	2 (4%)
	Medical director	1 (2%)
**Experience level**	Resident (novice)	21 (42%)
	Experienced	29 (58%)
**Years of experience**	0 - 3 years	13 (26%)
	3 - 5 years	7 (14%)
	5 -10 years	12 (24%)
	> 10 years	18 (36%)

Supplementary Table 1. Demographic data of survey respondents. N = 50 surgeons from German clinics from the following specialities participated in the study: general and visceral surgery, thoracic surgery, urology, gynecology, neurosurgery, orthopedics, and transplant surgery.

	Number of items	AVE	CR	Cronbach's alpha	**Mean | Std** Agreement	**Mean | Std** Frequency
**Human factors**						
Language barriers	3	0.61	0.82	0.66	3.61 | 0.63	2.84 | 0.76
Basic physical needs	4	0.56	0.83	0.73	3.82 | 0.61	2.96 | 0.60
Emotional stress	4	0.63	0.87	0.79	4.25 | 0.59	3.30 | 0.70
**Infrastructural challenges**						
Staff shortage	4	0.67	0.89	0.80	4.55 | 0.51	3.92 | 0.66
Lack of prior knowledge	3	0.59	0.80	0.64	3.95 | 0.58	3.42 | 0.64
Lack of standardization	3	0.82	0.93	0.88	4.21 | 0.80	3.62 | 0.72

Supplementary Table 2. Assessment of reliability, convergent validity and study results. To assess convergent validity, we analyzed whether the latent variables exceeded the recommended threshold of 0.5 for average variance extracted (AVE), while also ensuring that both composite reliability (CR) and Cronbach's alpha surpassed the threshold of 0.7. According to our results, Cronbach's alpha for items measuring the impact of language barriers and lack of prior knowledge is below the threshold of 0.7. However, above 0.6 is considered appropriate for exploratory research.

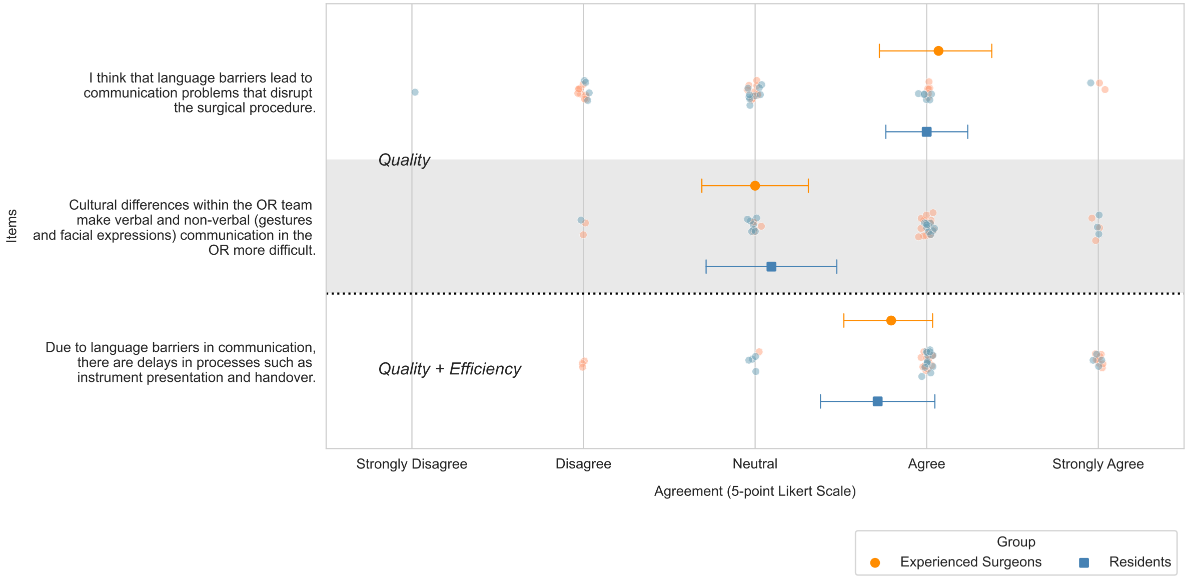

Supplementary Figure 1. Surgeon agreement ratings of human factors related challenges: Language barriers. $n_{experienced} = 29$; $n_{novice} = 21$; error plots outlining mean value and respective standard deviation.

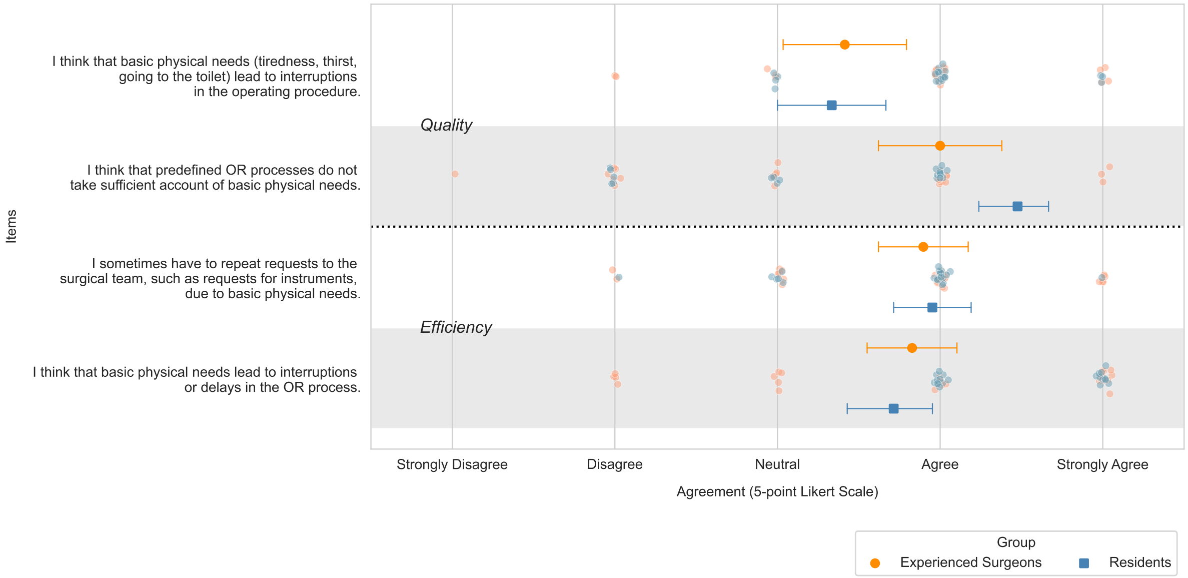

Supplementary Figure 2. Surgeon agreement ratings of human factors related challenges: Basic physical needs. $n_{experienced} = 29$; $n_{novice} = 21$; error plots outlining mean value and respective standard deviation.

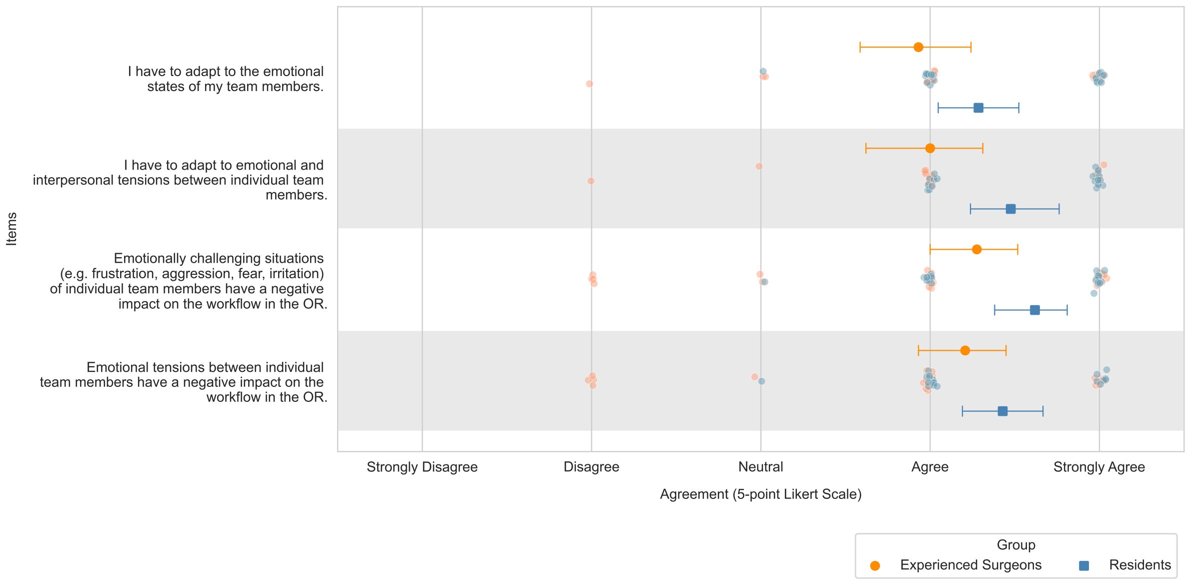

Supplementary Figure 3. Surgeon agreement ratings of human factors related challenges: Emotional stress. $n_{experienced} = 29$; $n_{novice} = 21$; error plots outlining mean value and respective standard deviation.

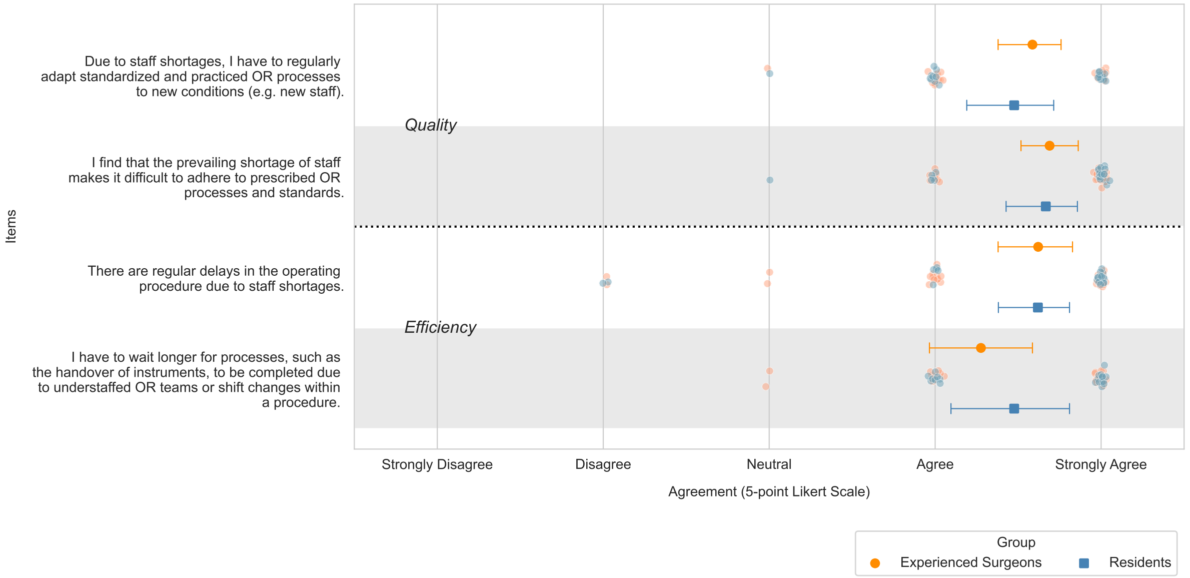

Supplementary Figure 4. Surgeon agreement ratings of infrastructural challenges: Staff shortage. $n_{experienced} = 29$; $n_{novice} = 21$; error plots outlining mean value and respective standard deviation.

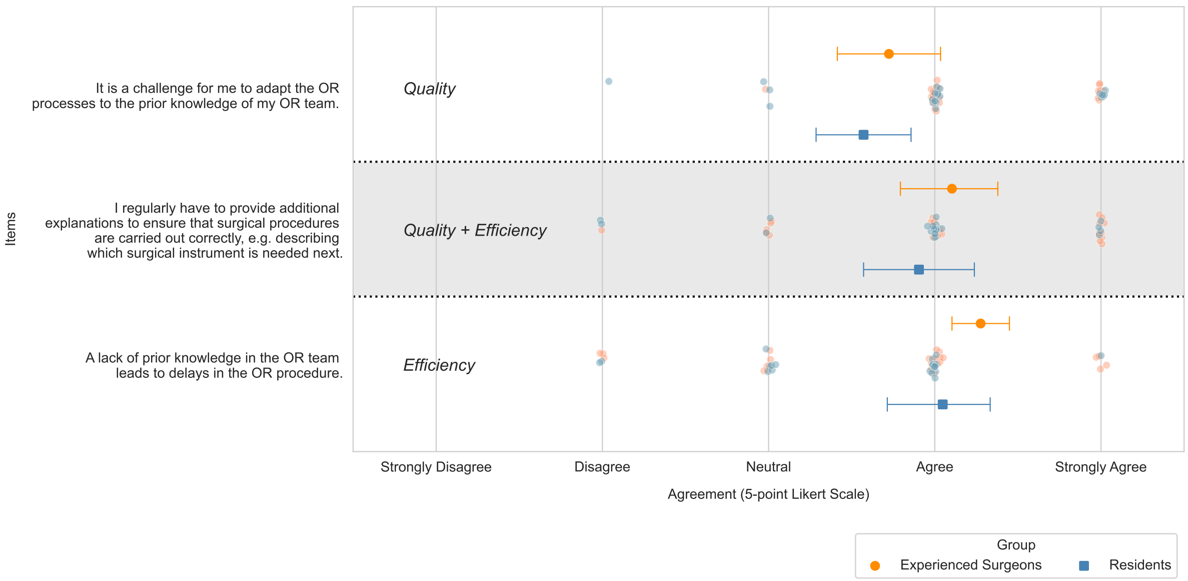

Supplementary Figure 5. **Surgeon agreement ratings of infrastructural challenges: Lack of prior knowledge.** $n_{experienced} = 29$; $n_{novice} = 21$; error plots outlining mean value and respective standard deviation.

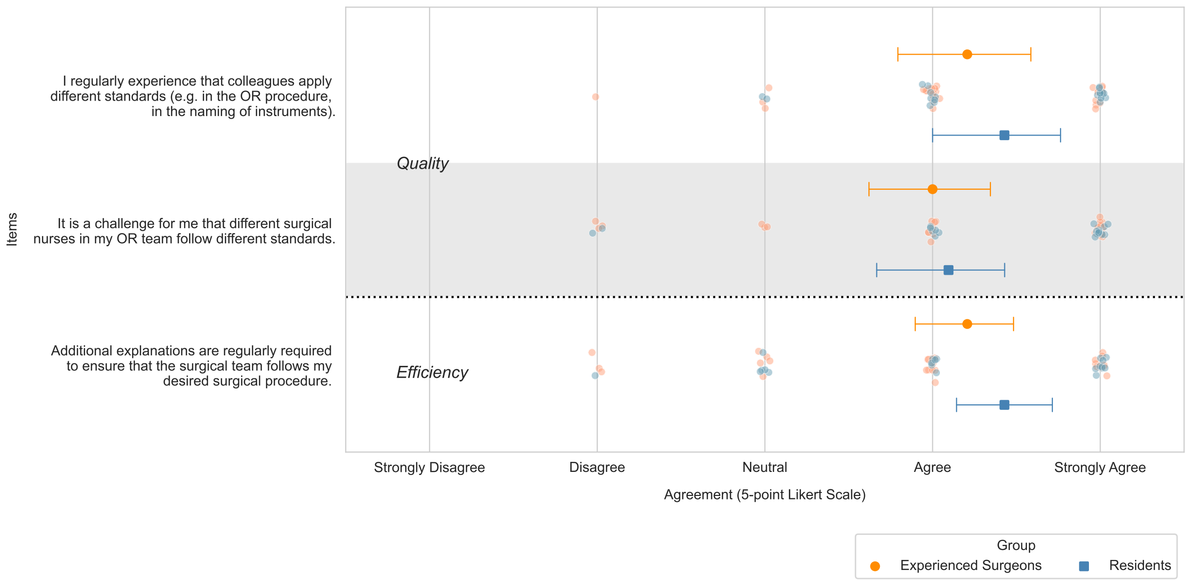

Supplementary Figure 6. **Surgeon agreement ratings of infrastructural challenges: Lack of process standardization.** $n_{experienced} = 29$; $n_{novice} = 21$; error plots outlining mean value and respective standard deviation.

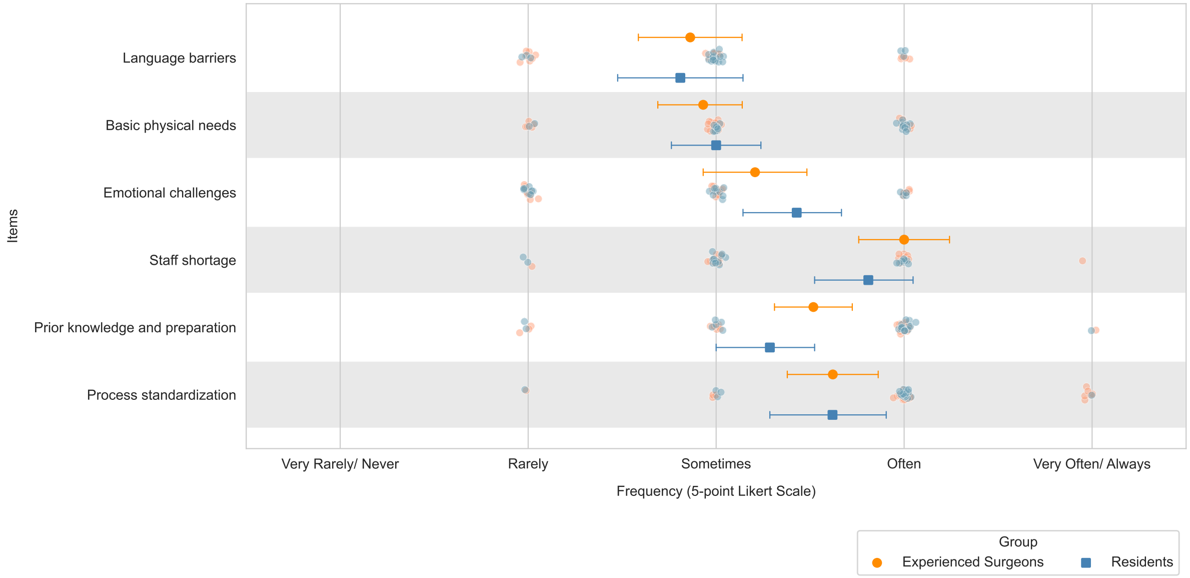

Supplementary Figure 7. Surgeon frequency ratings of human factors related and infrastructural challenges. $n_{experienced} = 29$; $n_{novice} = 21$; error plots outlining mean value and respective standard deviation.

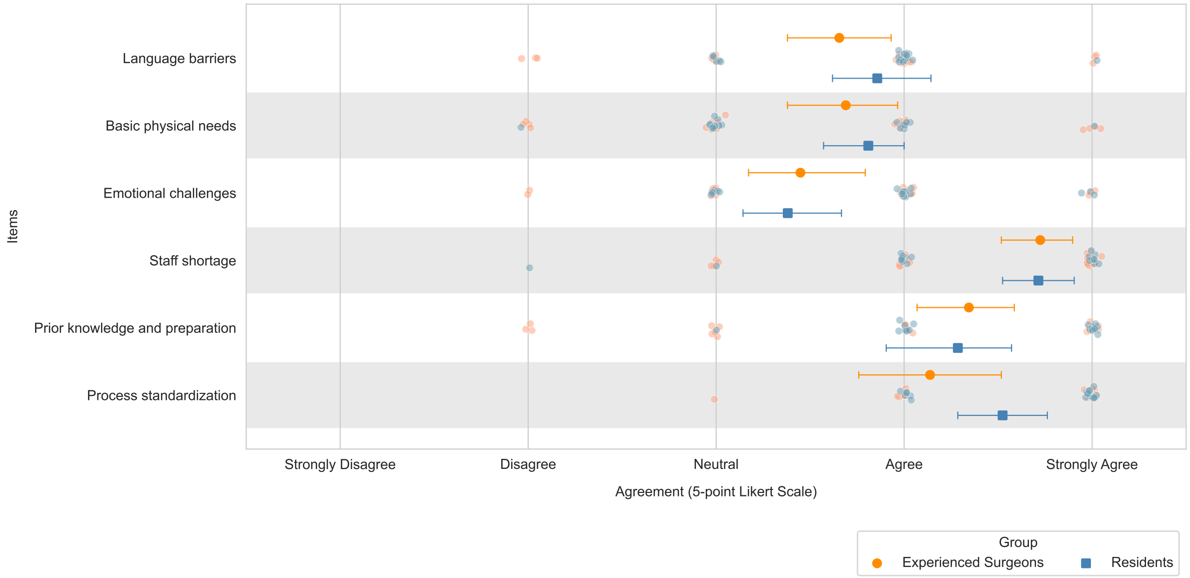

Supplementary Figure 8. Surgeon agreement ratings on the impact of human factors related and infrastructural challenges on the efficiency of OR workflows. "The following factors have a negative impact on the efficiency of OR processes and lead to delays"; $n_{experienced} = 29$; $n_{novice} = 21$; error plots outlining mean value and respective standard deviation.

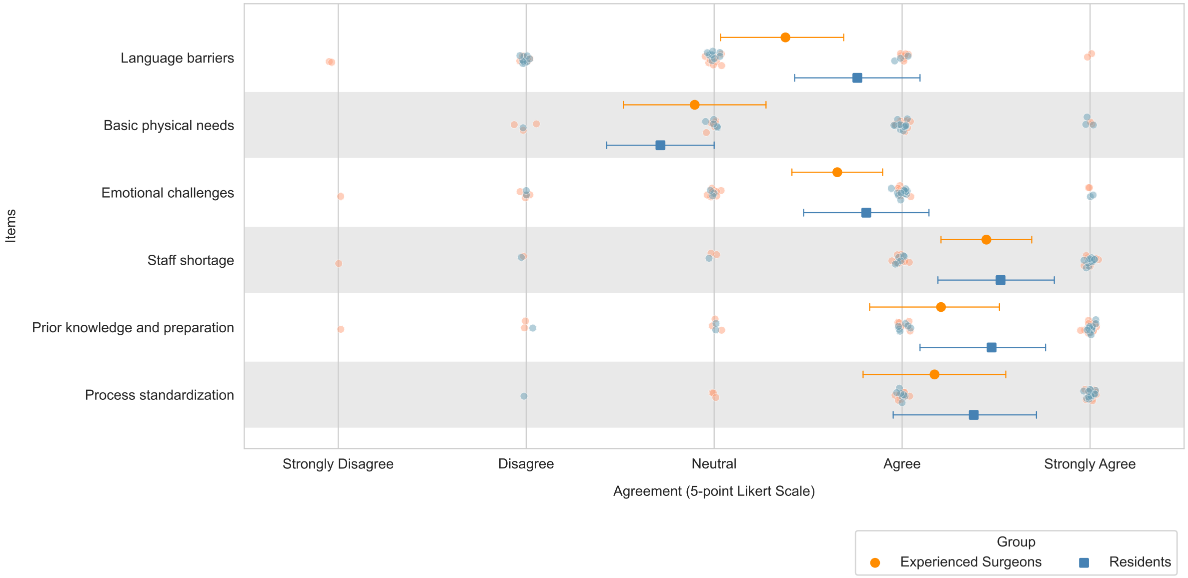

Supplementary Figure 9. Surgeon agreement ratings on the impact of human factors related and infrastructural challenges on the quality of OR workflows. "The following factors have a negative impact on the quality of surgical procedures and lead to a higher risk for the patient"); $n_{experienced} = 29$; $n_{novice} = 21$; error plots outlining mean value and respective standard deviation.

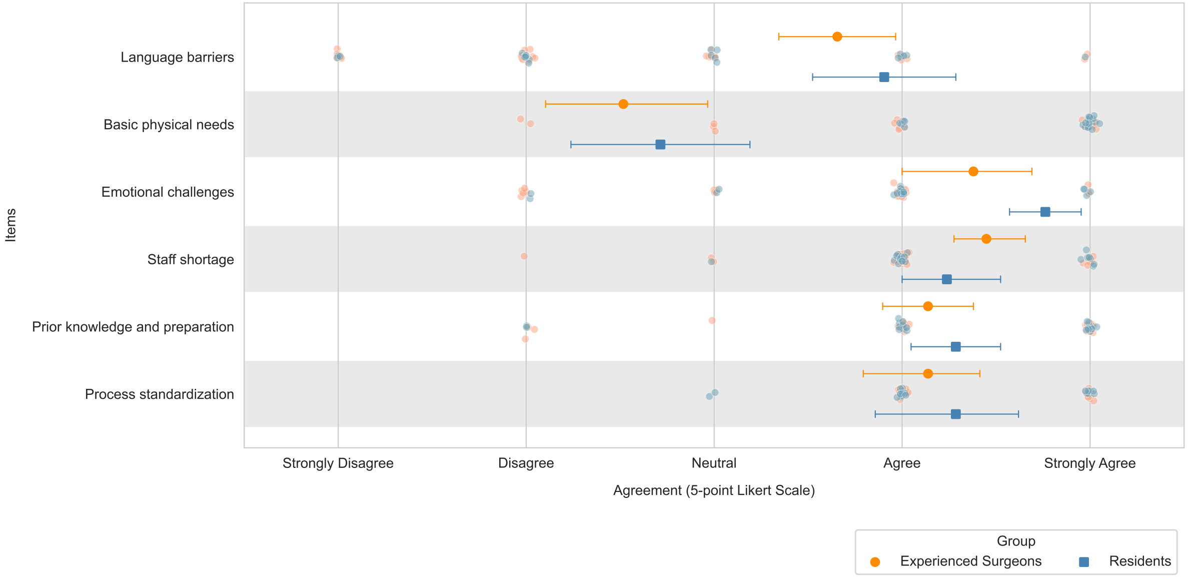

Supplementary Figure 10. Surgeon agreement ratings on the impact of human factors related and infrastructural challenges on the OR team work. "The following factors have a negative impact on teamwork within the OR team"; $n_{experienced} = 29$; $n_{novice} = 21$; error plots outlining mean value and respective standard deviation.

Trocar	mAP	mAR	mAA	mAF1
Right	60.37 ± 3.08	57.34 ± 2.16	91.21 ± 0.35	55.37 ± 1.94
Left	96.10 ± 1.26	95.73 ± 1.83	94.66 ± 1.83	94.62 ± 1.93

Supplementary Table 3. Macro-averaged evaluation metrics for the two working trocars. The evaluation metrics are averaged instrument-wise in the test data set, namely macro-averaged precision (mAP), recall (mAR), accuracy (mAA), and f1 score (mAF1). The averaged metrics over 10 folds are reported (%) with the corresponding standard deviation (±).

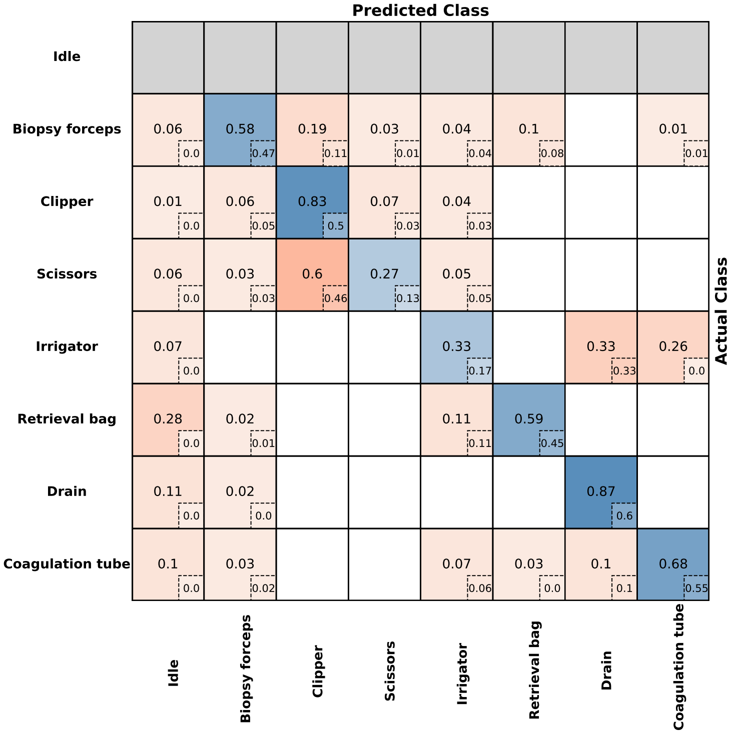

Supplementary Figure 11. Multi-class confusion matrix of surgical instruments for the right trocar. The matrix compares the predicted classes (idle, biopsy forceps, clipper, scissors, irrigator, retrieval bag, drain, coagulation tube) against the actual classes. Each cell shows the row-wise normalized proportion of predictions, with values ranging from 0 to 1, showing the f-Recall for each class. Diagonal cells represent correct predictions, with darker shades indicating higher values. The matrix reveals varying levels of classification performance across different surgical instruments.

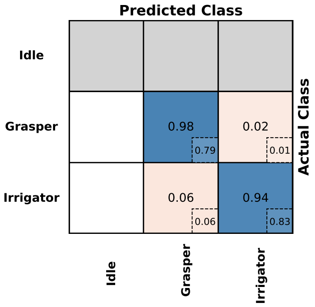

Supplementary Figure 12. Multi-class confusion matrix of surgical instruments for the left trocar. The matrix compares the predicted classes (idle, grasper, irrigator) against the actual classes. Each cell shows the row-wise normalized proportion of predictions, with values ranging from 0 to 1, showing the f-Recall for each class.
