# Supplementary material for: Robotic scrub nurse to anticipate surgical instruments based on real-time laparoscopic video analysis
Source: Commun Med (Lond). 2024 Aug 2;4:156. doi: 10.1038/s43856-024-00581-0 (PMC11297199; doi:10.1038/s43856-024-00581-0)
Supplement: Supplementary file 3 — Description of Additional Supplementary Files [file 43856_2024_581_MOESM3_ESM.pdf]

Description of Additional Supplementary Files

File name- Supplementary Data 1

File description- Figure 1 data of the main text.

File name- Supplementary Data 2

File description- Table 1 and 2 data of the main text.
